# Metastasectomy Following Immunotherapy with Adoptive Cell Transfer for Patients with Advanced Melanoma

**DOI:** 10.1245/s10434-016-5537-0

**Published:** 2016-09-16

**Authors:** Nicholas D. Klemen, Paul L. Feingold, Stephanie L. Goff, Marybeth S. Hughes, Udai S. Kammula, James C. Yang, David S. Schrump, Steven A. Rosenberg, Richard M. Sherry

**Affiliations:** Surgery Branch, National Cancer Institute, National Institutes of Health, Bethesda, MD USA

## Abstract

**Background:**

Immunotherapeutic treatment strategies including adoptive cell transfer (ACT) for metastatic melanoma are capable of mediating complete and durable responses, as well as partial responses and prolonged disease stabilization. Unfortunately, many patients ultimately develop progressive disease. The role of salvage metastasectomy in managing these patients has not been evaluated.

**Methods:**

Records of patients with metastatic melanoma treated with ACT at a single institution between 2000 and 2014 were reviewed. Patients with an objective response by RECIST criteria or disease stabilization of at least 6 months and who subsequently developed progressive melanoma and were managed with metastasectomy as the next therapeutic strategy were studied for progression-free survival (PFS) and overall survival (OS). Five additional clinical parameters were also reviewed for association with outcomes.

**Results:**

Of 115 patients treated with ACT who met our response criteria and then developed progressive disease, 26 (23%) had surgery. There were no mortalities related to surgical intervention. Median follow-up after surgery was 62 months. Median PFS after surgery was 11 months and five-year OS was 57%. The development of a new site of metastasis after ACT was associated with poor PFS and OS.

**Conclusions:**

Surgery after immunotherapy is safe. Long PFS and OS can be achieved by metastasectomy in selected patients with progressive melanoma following treatment with ACT. Clinical variables important for patient selection for metastasectomy after immunotherapy remain largely undefined. Improvements in immunotherapeutic treatment strategies may increase the role of surgery for patients with advanced disease.

Recent advances in immunotherapy using adoptive cell transfer (ACT) of autologous lymphocytes^1^–^7^ and checkpoint inhibitors^8^–^11^ have dramatically altered the therapeutic landscape for patients with advanced melanoma. These immune-based strategies can mediate complete and durable responses, as well as partial responses (PRs) and prolonged disease stabilization. Unfortunately, many patients ultimately develop progressive melanoma after treatment. More specifically, the complete response (CR) rate following ACT has been reported to be 24 %, while another 30 % of patients experienced a PR, with an overall response rate of approximately 55 %. In this setting, CRs are almost always durable, but approximately 70 % of patients with only a PR subsequently developed progressive disease.^7^ For these patients, metastasectomy can be considered if tumor progression is limited.^12^,^13^

This report describes our experience using metastasectomy as the next treatment for a selected cohort of patients who developed progressive melanoma after having achieved an objective response or disease stabilization for at least 6 months following ACT. We primarily studied progression-free survival (PFS) and overall survival (OS) after salvage metastasectomy, and also studied five clinical variables to test whether any were associated with outcomes in this highly selected group of patients.

## Methods

### Patient Selection Criteria and Clinical Protocols

Our intent was to investigate the impact of metastasectomy for patients with advanced melanoma who had a clinical response to ACT given at the Surgery Branch, National Cancer Institute (NCI). To be included in this analysis, patients were required to have achieved an objective PR or CR according to Response Evaluation Criteria in Solid Tumors (RECIST) 1.0.^14^ We also included patients with stable disease (SD) documented for at least 6 months (SD6) following ACT as this appears to be an important and reproducible outcome following immunotherapy.^15^ Following an initial clinical response, all patients in this study had documented disease progression and underwent metastasectomy as the next treatment modality. We excluded patients who underwent surgery for research or palliation, and those who had resection of sites of prolonged SD without overt progression. Finally, patients with progressive brain metastases were excluded because they almost always require an intervention. All patients had viable melanoma confirmed in the surgical specimen.

### Adoptive Cell Transfer Protocols

Patients in this analysis had a diagnosis of metastatic melanoma and were enrolled in one of 11 Institutional Review Board (IRB)-approved clinical protocols transferring autologous tumor-infiltrating lymphocytes (TILs) or peripheral blood lymphocytes genetically modified to express a human leukocyte antigen (HLA)-appropriate T cell receptor (GM-PBL). Patients were required to have measurable metastatic disease and an Eastern Cooperative Oncology Group (ECOG) score of 1 or 2. The production, selection, and administration of these cells have previously been described in detail and the results of these clinical trials have been previously published.^2^,^6^ In brief, TILs (376 patients) were generated from a resected melanoma tumor deposit cultured in high-dose interleukin (IL)-2. TILs with sufficient growth and/or demonstrable anti-tumor reactivity were expanded for treatment. The GM-PBL (76 patients) was generated by retroviral transduction of autologous peripheral blood lymphocytes with a T-cell receptor recognizing one of four different HLA-A*02 restricted melanoma antigens (Mage-A3, Mart-1, gp-100(154), or NY-ESO).

Prior to lymphocyte infusion, all patients received lymphodepleting, but non-myleoablating, chemotherapy consisting of 2 days of cyclophosphamide (60 mg/kg/day) followed by 5 days of fludarabine (25 mg/m^2^/day). A subset of TIL patients also received total body irradiation (2 or 12 Gy fractionated over 3 days), along with autologous CD34^+^ stem cell support. Approximately 4 h after cell infusion, all patients were started on high-dose IL-2 at 720,000 IU/kg every 8 h as tolerated.

### Protocol Surveillance and Preoperative Evaluation

Patients on ACT protocols had scheduled evaluations, which included computed axial tomography (CT) of the chest, abdomen, and pelvis before and within 4 weeks of ACT and then monthly for 3 months. Patients with hepatic disease were evaluated using magnetic resonance imaging (MRI). Other imaging modalities were added as needed to evaluate specific disease sites. For each protocol, patients were evaluated every 2 months with complete imaging for the remainder of the first year. Subsequently, imaging was obtained at 3- to 6-month intervals for up 5 years. MRI evaluation of the brain was required prior to protocol enrollment and subsequently obtained every 4–6 months. Patients undergoing metastasectomy had complete preoperative imaging, including brain MRI. Positron emission tomography (PET) scans were not used for tumor response evaluations but were obtained preoperatively at the discretion of the operating surgeon. This intensive radiologic surveillance allowed sites of disease progression to be retrospectively classified as either a pre-existing site or a new site of disease, relative to the start date of ACT.

Postoperative evaluations included repeat imaging and were obtained at 3- to 4-month intervals for the first year and then at 6-month intervals for up to 5 years. A brain MRI was typically obtained every other visit (or every visit for those with a history of central nervous system [CNS] disease). Although some patients who developed progressive melanoma following surgical resection underwent repeat metastasectomy, most patients were either enrolled in other investigational protocols or discharged from the Surgery Branch, NCI. Consequently, the overall survival data for these patients may have been impacted by subsequent therapies.

### Surgical Approach

The goal of the metastasectomy was to eliminate all sites of *progressing* melanoma. Importantly, metastases that were stable or still shrinking following cell therapy were not necessarily resected. Patients left with no evidence of disease (NED) after surgery had no residual abnormalities identified by imaging or physical examination. Patients with stable or regressing disease after surgery were considered to have residual disease.

### Statistical Analysis

We measured PFS and OS from the date of metastasectomy using the Kaplan–Meier method. Patients with planned multistage operations were counted from the date of the first procedure. We studied five clinical variables for an association with postoperative outcomes using the log-rank method. Three of these variables have been reported to be associated with favorable outcomes in reported series of metastasectomy for melanoma, and include resection of solitary or multiple tumors, postoperative disease status (NED or residual disease), and resection of visceral or non-visceral tumors. The disease-free interval has also been considered to be an important prognostic factor, and consequently we included the interval between ACT and metastasectomy (<12 months or >12 months) as a factor to be analyzed. The fifth clinical variable was whether the resected lesion was present prior to ACT (pre-existing site) or only became apparent after ACT (new site). We included this variable because of the discrepancy between RECIST and the immune-related response criteria (irRC) for the determination of objective anti-tumor responses. A new tumor site is considered progressive disease using RECIST criteria but is added to the overall tumor burden when irRC are used.^14^,^16^ Due to the exploratory nature of this retrospective study and the small size of the study cohort, we did not perform a Bonferroni correction. We planned to generate a multivariate model in the event that two or more variables reached a threshold of *p* ≤ 0.1.

## Results

**Fig. 1:**
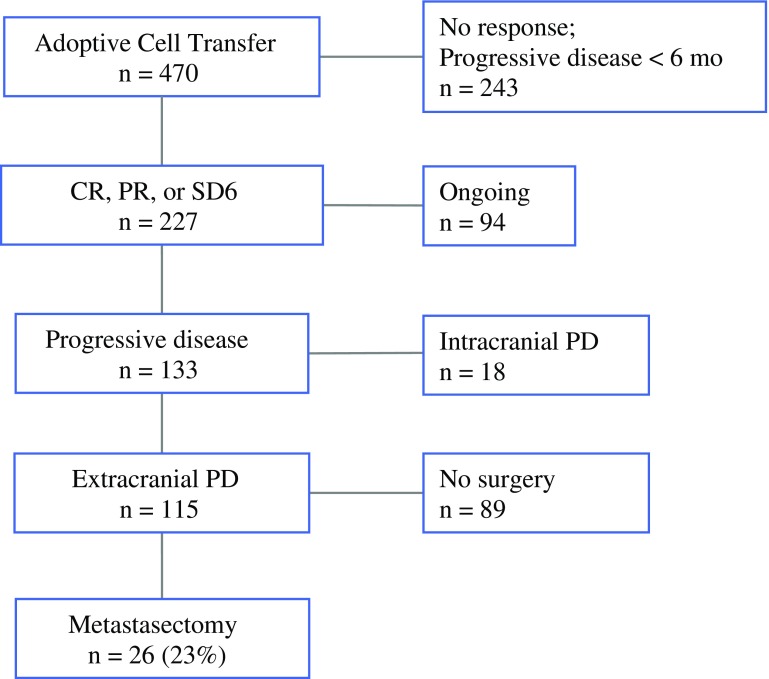
Patient selection process for metastasectomy after ACT. *CR* complete response, *PR* partial response, *SD6* stable disease documented for at least 6 months, *PD* progressive disease

A total of 470 patients with advanced cutaneous melanoma were enrolled in one of the 11 ACT protocols from 2000 to 2014 (Fig. 1). Overall, 227 patients achieved an objective response or SD for at least 6 months. Although the responses were durable for 94 patients, 133 patients developed progressive melanoma, 18 of whom developed brain metastasis and required craniotomy or radiation therapy and were subsequently excluded from this analysis. Twenty-six of the remaining 115 patients (23 %) with extracranial progressive melanoma underwent metastasectomy; this highly selected cohort represents the focus of our study.

**Fig. 2:**
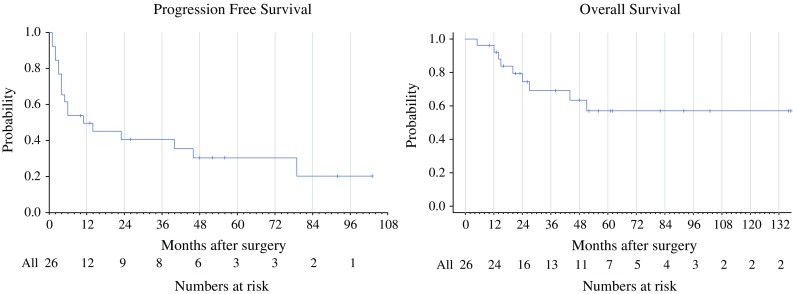
Kaplan–Meier curves showing progression-free survival and overall survival (OS) after metastasectomy (*n* = 26)

Prior to treatment by ACT, 19 of the 26 patients eventually undergoing metastasectomy had M1c disease (Tables 1, 2). These 26 patients had a median number of five metastatic lesions per patient, with a range of 2–23 lesions. There were no mortalities related to metastasectomy. With a median follow-up after metastasectomy of 62 months, the median PFS was 11 months and the 5-year OS was 57 % for all 26 patients (Fig. 2). After surgery, nine patients (35 %) had a PFS of more than 24 months, and six patients (23 %) had a PFS of more than 46 months.

**Table 1:** Demographics of the study cohort before ACT. *ACT* adoptive cell transfer

Demographics before ACT	
Men, women	17, 9
Median age of ACT	49
M stage at the time of ACT	M1a = 4
	M1b = 3
	M1c = 19
Number of metastases at the time of ACT	Median = 5
	Average = 6
Previous therapies	Interferon = 12
	Vaccination = 2
	Chemotherapy = 4
	Interleukin-2 = 15
	Ipilimumab = 2
	ACT = 3

**Table 2:** Characteristics of the study cohort

M stage and metastases correspond to the stage and number of metastatic lesions known before ACT. ‘Response’ indicates the response to ACT (using RECIST); ‘Interval’ indicates the time (in months) between ACT and metastasectomy; *blue shading* indicates patients with ongoing PFS and OS after metastasectomy; *green shading* indicates patients with ongoing OS after progressive disease; and *red shading* indicates patients who expired with disease

*ACT* adoptive cell transfer, *RECIST* Response Evaluation Criteria in Solid Tumors, *PFS* progression-free tumor, *OS* overall survival, *LDH* lactate dehydrogenase, *PR* partial response, *SD* stable disease, *CR* complete response, *LN* lymph node, *NED* no evidence of disease, *N/A* not applicable, *WBRT* whole brain radiotherapy

^a^Indicates elevated serum LDH at the time of metastasectomy

^b^Indicates incomplete resection of intended progressing lesions

^c^Indicates ongoing PFS or OS

We found 8 of the 26 patients undergoing metastasectomy remained progression-free and required no additional therapy (Table 2). For the remaining patients, subsequent therapies are noted. Interestingly, three additional patients have achieved prolonged survival following only repeat metastasectomy, and two patients have survived for over 10 years following re-treatment with ACT. The remaining three long-term survivors were later treated with checkpoint inhibitors.

### Clinical Factors Associated with Favorable Progression-Free Survival

**Fig. 3:**
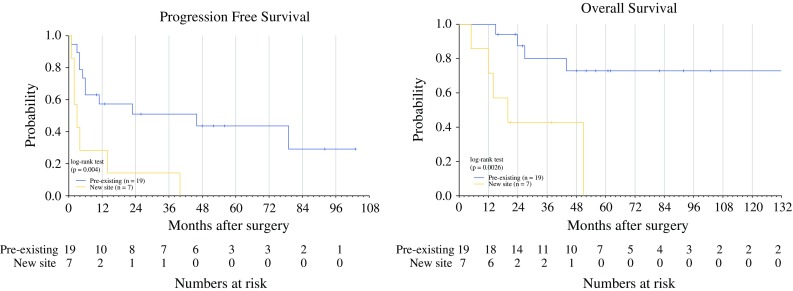
Kaplan–Meier curves showing progression-free survival and overall survival of patients after resection of a pre-existing tumor (*n* = 19; *blue line*) or a new tumor (*n* = 7; *yellow line*). Univariate analysis was performed using the log-rank test

Univariate analyses revealed no significant PFS or OS associations between non-visceral resections, solitary sites of resection, and NED status after surgery (Table 3). An interval between ACT and metastasectomy of >12 months was also not associated with improved PFS or OS. We did observe that longer PFS and OS were significantly associated with the resection of a pre-existing site of metastasis compared with a new site of disease (Fig. 3). Median PFS for these patients was 46 months and 3 months, respectively (*p* = 0.004). The 5-year OS rates following surgery on pre-existing lesions versus new lesions were 73 and 0 % respectively.

**Table 3:** Clinical factors analyzed for association with PFS and OS after metastasectomy

Univariate	Number of patients	Median PFS (Months)	Median OS (Months)	Five-year OS
Interval between ACT, surgery		*p* = 0.34	*p* = 0.65	
<1 year	12	5	NR	60 %
>1 year	14	23	51	49 %
Number of tumors resected		*p* = 0.31	*p* = 0.58	
One	18	23	NR	56 %
Two	8	3.5	44	47 %
Location of tumors		*p* = 0.17	*p* = 0.43	
Nodal/subcutaneous	9	4	NR	53 %
Visceral (or elevated LHD)	17	40	NR	61 %
Post-op disease status		*p* = 0.82	*p* = 0.29	
No evidence of disease	18	14	NR	62 %
Residual disease	8	7.5	27	45 %
Type of metastasis		*p* = 0.004	*p* = 0.003	
Pre-existing site	19	46	NR	73 %
New site	7	3	20	0 %

*PFS* progression-free survival, *OS* overall survival, *ACT* adoptive cell transfer, *LDH* lactate dehydrogenase, *NR* not reported

## Discussion

Metastasectomy in highly selected patients with advanced melanoma is widely accepted and can potentially be curative. Data to support the resection of metastatic melanoma for curative intent has largely been limited to retrospective series from single institutions.^17^–^20^ Proper patient selection has long been recognized to be critical to achieving optimal surgical results. Clinical factors previously shown to be associated with favorable PFS and OS include resection of solitary tumors, non-visceral tumors, complete resection, and prolonged disease-free interval.

There are few data describing the outcomes of metastasectomy after immunotherapy. One series reported that metastasectomy did not convincingly achieve prolonged PFS for patients with progressive melanoma who responded to high-dose IL-2.^12^ Conversely, another study noted that patients with metastatic melanoma made free of disease by thoracic resection had a 5-year actuarial survival rate of 76 %; the majority of these patients had been treated with IL-2, vaccine, or interferon.^13^ To our knowledge, the reported experience with metastasectomy for metastatic melanoma following checkpoint inhibition is limited to a single report. Gyorki et al. reported a median PFS of 9.5 months after metastasectomy in patients being treated with ipilimumab.^21^

Five-year survival following therapy with ipilimumab has been reported to range from 17 to 38 %, and, following ACT, has been reported to be over 40 %.^15^ Response rates to anti-PD1 antibodies are improved over ipilimumab, and have shown efficacy in other histologies.^11^,^22^ The ability of these immune-based strategies to mediate complete and durable tumor regression along with prolonged disease stabilization prompted us to evaluate our experience using metastasectomy as the next treatment option for patients following ACT. This series is interesting because all 26 patients had multiple lesions and widespread disease, which made them not ideal candidates for curative surgery before ACT.^19^ For example, before ACT no patient had a solitary metastasis, and the four patients with two metastases had M1c disease. All remaining patients had three or more metastatic lesions, with a median of five per patient. The option of metastasectomy was reconsidered only after a strong response to immunotherapy, which was often associated with the resolution of multiple tumors.

Consistent with all series reporting metastasectomy for patients with advanced melanoma, our study consisted of a highly selected population. However, for patients who achieved an objective response or had SD for at least 6 months, metastasectomy was used for 23 % of patients with extracranial disease progression after ACT. The fact that 9 of the 26 ACT patients achieved a PFS of over 24 months and 6 patients achieved a PFS of over 46 months after a single operation confirms that selected patients relapsing after a response to immunotherapy can safely undergo an effective salvage operation. Because appropriate patient selection is both critical and difficult, we also sought to identify clinical features that might predict patients who were likely to benefit from surgical resection. This study is an exploratory analysis of a small cohort; consequently a Bonferroni correction was not performed. The small numbers of patients put it at high risk for type II error. Even still, the only variable significantly associated with favorable PFS and OS after surgery was if pre-existing sites of disease were resected versus new sites that appeared after immunotherapy. Of note was the fact that stable or still shrinking sites of disease were not resected in some patients, yet PFS and OS that was similar to patients who were made disease free. Our experience suggests that stable or still responding sites of metastatic melanoma may not need to be resected following a response to ACT.

As previously noted, Gyorki et al. described the Memorial Sloan Kettering Cancer Center experience with 23 patients undergoing surgery for metastatic melanoma while receiving ipilimumab, and noted that 3 patients remained free of disease and 10 were alive with disease. The report by Gyorki et al., combined with our findings, prompted us to review our experience with metastasectomy following treatment with ipilimumab using the same criteria that were described in the Methods section herein. We had previously reported our experience with 177 patients treated with ipilimumab for metastatic melanoma.^10^ From this group, 32 patients met our response criteria and progressed at extracranial sites. Ultimately, 6 of these 32 patients (19 %) underwent metastasectomy as the next treatment, and 3 have remained disease-free for over 5 years. Interestingly, these long-term survivors were made NED with surgery and had resection of pre-existing sites of disease.

## Conclusions

Our experience suggests that long PFS can be achieved by metastasectomy in selected patients with progressive melanoma following treatment with ACT. Approximately 23 % of patients with a CR, PR, or SD6 before subsequent tumor progression were candidates for salvage metastasectomy. Patients who had resection of pre-existing tumors had favorable PFS and OS compared with patients undergoing resection of new disease sites. Due to the retrospective and exploratory design of this study, these findings will need to be evaluated by other institutions with larger numbers of patients. Finally, it will be important to determine if our experience with salvage metastasectomy following ACT offers insights for the management of patients with melanoma who develop tumor progression following a response to checkpoint inhibitors.
